# Addressing Sexual and Gender Diversity Through Inclusive Pedagogical Practices in Global Preregistration Nursing and Midwifery Education: Protocol for a Scoping Review

**DOI:** 10.2196/93408

**Published:** 2026-09-14

**Authors:** Lucille Kelsall-Knight, Jennifer M Goldberg, Kelvin McMillan, Elizabeth K Darling, Charlene Downing

**Affiliations:** 1 University of Birmingham Birmingham, England United Kingdom; 2 McMaster University Hamilton, ON Canada; 3 University of Johannesburg Johannesburg, Gauteng South Africa

**Keywords:** sexual and gender minority groups, lesbian, gay, bisexual, transgender, queer or questioning, intersex, asexual, and other sexual and gender identities, LGBTQIA+, gender identity, sexual orientation, inclusive education, nursing education, midwifery education, curriculum, teaching methods, simulation training, faculty development, health equity, cultural humility, intersectionality, scoping review

## Abstract

**Background:**

Sexual and gender diverse populations experience inequities in health and health care access, as well as gaps in health professional education. Preregistration nursing and midwifery education represents a critical formative stage for developing inclusive and affirming practice; however, the integration of sexual and gender diversity within curricula remains variable and inconsistently described. Mapping how inclusive pedagogical practices address sexual and gender diversity in preregistration nursing and midwifery education will clarify current approaches and identify gaps in the literature.

**Objective:**

This study aims to map how sexual and gender diversity is addressed through inclusive pedagogical practices in preregistration nursing and midwifery education globally.

**Methods:**

This protocol follows the Joanna Briggs Institute (JBI) guidance for scoping reviews and the PRISMA-ScR (Preferred Reporting Items for Systematic Reviews and Meta-Analyses Extension for Scoping Reviews) reporting checklist and will be reported in accordance with PRISMA-ScR guidance. We will search the CINAHL, MEDLINE, PubMed, Scopus, and Web of Science databases, as well as targeted gray literature. Two reviewers will independently screen titles and abstracts, followed by full-text screening, and extract data using a piloted form through Covidence. Prior to formal screening, reviewers will pilot-test the inclusion criteria on a sample of titles and abstracts to ensure consistency. Agreement will be discussed, and the eligibility criteria will be refined as required. Data extraction will include pedagogical practices addressing sexual and gender diversity (teaching strategies, curriculum content, simulation, and assessment), theoretical or conceptual frameworks; outcomes and indicators (knowledge, attitudes, skills, confidence, and learning environment), and any identified equity considerations (attention to gender identity and sexual orientation and intersectionality). Synthesis and presentation of findings will be completed using the Patterns, Advances, Gaps, Evidence for Practice, and Research Recommendations (PAGER) framework.

**Results:**

The scoping review was initiated in May 2026 following protocol registration with the Open Science Framework. Preliminary searches conducted in November 2025 informed the final search strategy. The final searches were conducted in May 2026 across 5 databases. At the time of manuscript submission, screening and data extraction were in progress. Data charting and synthesis are expected to be completed by October 2026, with findings anticipated for dissemination in December 2026.

**Conclusions:**

This review is expected to identify diverse but inconsistently applied inclusive pedagogical practices, which show a predominance of short-term outcome evaluations and limited theoretical underpinning. It will also highlight key gaps, particularly in the inclusion of gender diverse populations, midwifery-specific evidence, and rigorously evaluated interventions. This review will provide a comprehensive map of inclusive pedagogical practices addressing sexual and gender diversity in preregistration nursing and midwifery education, identify gaps in the evidence base, and inform curriculum development and educator training.

**Trial Registration:**

Open Science Framework 10.17605/OSF.IO/8GHVD; https://osf.io/8ghvd

**International Registered Report Identifier (IRRID):**

PRR1-10.2196/93408

## Introduction

### Overview

Inclusive pedagogical practices are critical to preparing nursing and midwifery students to deliver equitable, affirming care for sexual and gender diverse (SGD) populations [1,2]. For the purpose of this review, inclusive pedagogical practices are understood as intentional teaching, learning, assessment, and curriculum design approaches that actively recognize, affirm, and respond to sexual and gender diversity; challenge heteronormativity and cisnormativity; and promote equitable participation and learning outcomes for all students. Sexual and gender diversity is conceptualized as encompassing diverse sexual orientations; gender identities and expressions; and sex characteristics, including but not limited to Two-Spirit, lesbian, gay, bisexual, transgender, queer or questioning, intersex, asexual, and other sexual and gender identities (2SLGBTQIA+). The term 2SLGBTQIA+ is used to reflect a broad and inclusive range of sexual orientations and gender identities. “Two-Spirit” is a Western term used by many Indigenous communities in Canada and the United States to describe Indigenous people of diverse gender identities, expressions and roles, and diverse sexual orientations. Approaches to incorporating sexual and gender diversity within preregistration curricula vary widely, encompassing content inclusion, instructional strategies, learning environments, assessment, and educator development [3,4]. A scoping review is the appropriate methodology to map the breadth and nature of evidence across diverse study designs, educational settings, and sources and to identify key concepts and gaps that may inform systematic reviews or guideline development.

### Background

SGD populations continue to experience significant inequities in health and health care access, driven by structural stigma, discrimination, and gaps in health care professional knowledge and confidence. International evidence demonstrates that SGD communities frequently report negative encounters with health professionals, including assumptions grounded in heteronormativity and cisnormativity, lack of understanding of diverse identities, and limited recognition of their specific health needs [1,2,5]. These findings are echoed in studies documenting that lesbian, gay, bisexual, and transgender (LGBT) parents and lesbian‑parented families often encounter heterocentric documentation, heterosexist assumptions, and discriminatory attitudes when accessing health care [6-9]. These experiences contribute to avoidable disparities in mental health, chronic illness management, reproductive health, and access to preventative services. As frontline practitioners, nurses and midwives play a central role in shaping health care experiences. Preregistration education represents a critical formative stage in which students develop foundational knowledge, attitudes, and professional identities. Focusing on this stage enables the examination of how inclusive pedagogical practices are embedded early in professional training. Ensuring that preregistration nursing and midwifery programs adequately prepare graduates to provide affirming, equitable care is therefore a critical educational and public health priority.

Despite the growing recognition of the need for inclusive and affirming education, research indicates considerable variability in how sexual and gender diversity is embedded in health professional training [3,4]. Reviews and empirical work highlight that while many midwives hold positive attitudes toward sexual and gender minority clients, important gaps remain in training, confidence, and preparedness [10,11]. Some institutions integrate discrete content focused on SGD population health, while others adopt broader curricular strategies such as simulation‑based learning, reflective practice, case‑based teaching, or community engagement. However, educator‑reported barriers are common, with limited training, uncertainty, and a lack of institutional support for teaching sexual and gender diversity identified across health care professions, including midwifery [3,12,13]. This variability results in inconsistent learning experiences for students and may limit the development of essential competencies related to communication, cultural humility, and affirming clinical practice. Nursing and midwifery education are considered together because of their shared professional values related to person-centered care, communication, equity, and the preparation of practitioners to deliver inclusive care to diverse populations. However, important differences may exist in curriculum content, educational priorities, and program structures. Midwifery programs in some countries are delivered through direct-entry routes (such as Canada, New Zealand, and the United Kingdom), while others require prior nursing preparation (such as the United States). These contextual differences will be considered during data extraction and interpretation of findings, and profession-specific patterns will be highlighted where possible.

Inclusive pedagogical practices are increasingly promoted to address these gaps. Such practices extend beyond simply adding content; they involve intentional strategies to shape learning environments, design assessments, select teaching approaches, and challenge implicit norms embedded within curricula. Within nursing and midwifery education, inclusive pedagogy may include the integration of diverse case scenarios; explicit attention to clinical communication skills; the use of simulation involving scenarios relevant to SGD populations; structured learning about terminology and concepts; and approaches that encourage students to critically examine bias, norms, and structural determinants of health. Evidence from work on cultural humility in nursing underscores that reflective, relational, and equity‑oriented approaches can support more inclusive and person‑centered care [14,15]. Furthermore, insights drawn from transformative learning and caring science highlight their contribution to shaping students’ attitudes and professional identity, reinforcing the value of pedagogical approaches that promote reflection, inclusivity, and culturally responsive practice [16-18]. Nevertheless, the nature, scope, and effectiveness of these pedagogical practices remain unevenly described in the literature.

Given the breadth of approaches and the emerging nature of this field, scoping existing evidence is essential to guide curriculum development, inform educator training, and support institutions seeking to advance equity-focused educational practices. A scoping review offers a rigorous method for mapping how sexual and gender diversity is currently addressed within nursing and midwifery education, identifying theoretical or conceptual frameworks used, and highlighting gaps where further research or evaluation is needed [19,20]. Clarifying the current state of evidence will also provide a foundation for future systematic reviews and contribute to the development of best‑practice guidance for educators and program leaders.

Several cited studies represent the coauthors’ prior work, which has informed the conceptual development of this review; however, these are presented alongside a broader body of international literature to ensure balanced representation. The decision to focus on the literature published from 2021 onward reflects developments in equity, diversity, and inclusion initiatives within health professions education globally. Increasing recognition of systemic inequities in health care, growing attention to transgender- and gender diverse health needs, and calls from professional organizations for more inclusive curricula have accelerated educational innovation in this area. The review therefore seeks to map contemporary pedagogical approaches that reflect current understandings of inclusive practice rather than historical approaches that may no longer align with present educational and social contexts.

### Objectives and Review Questions

The primary review question is as follows: How are sexual and gender diversity addressed through inclusive pedagogical practices in preregistration nursing and midwifery education?

The secondary review questions are as follows: (1) What types of inclusive pedagogical practices are reported in preregistration nursing and midwifery education to address sexual and gender diversity, and how are these practices characterized and implemented? (2) What theoretical or conceptual frameworks underpin these pedagogical practices? (3) What gaps exist in the literature regarding inclusive pedagogy for sexual and gender diversity in nursing and midwifery education?

## Methods

### Protocol Registration and Reporting

This protocol is informed by the Joanna Briggs Institute (JBI) *Manual for Evidence Synthesis* guidance on scoping reviews and will be reported in accordance with the PRISMA‑ScR (Preferred Reporting Items for Systematic Reviews and Meta-Analyses Extension for Scoping Reviews) checklist. The completed PRISMA‑ScR checklist will be submitted with the protocol. This protocol has been registered on the Open Science Framework (OSF; 10.17605/OSF.IO/8GHVD [21]).

### Eligibility Criteria

#### Eligibility Framework (Population, Concept, and Context)

##### Population

This includes students enrolled in preregistration nursing and/or midwifery programs and the educators or academic staff involved in delivering relevant teaching.

##### Concept

This encompasses inclusive pedagogical practices addressing sexual and gender diversity (Two-Spirit, lesbian, gay, bisexual, transgender, queer or questioning, intersex, asexual, and other sexual and gender identities–affirming content; inclusive curriculum design; teaching and learning strategies; simulation; assessment; faculty development; classroom climate; and equity or antidiscrimination initiatives related to teaching and learning).

##### Context

This includes preregistration nursing and midwifery education delivered in higher education institutions or accredited training providers, across classroom, clinical skills or simulation, and practice-based learning environments.

#### Inclusion Criteria

The review will include evidence relating to preregistration nursing and midwifery students, as well as educators or academic staff involved in their teaching. While some jurisdictions offer midwifery education based on previous nursing preparation, only undergraduate midwifery (direct entry) programs are included in this review. Eligible sources must address explicit pedagogical practices concerning sexual and gender diversity, which may encompass curriculum content, teaching methods, learning activities, assessment approaches, simulation-based education, educator development initiatives, or broader learning environment interventions. Studies and materials will be drawn from higher education institutions or accredited training settings where preregistration programs are delivered, including classroom, skills or simulation laboratories, and practice-based learning environments. Empirical research of any design (quantitative, qualitative, or mixed methods) and evaluations providing sufficient pedagogical detail will be considered. Only materials published in English will be eligible, and the search will include publications from 2021 onward, with the final search date reported in the completed review. The year 2021 was selected to capture the most recent and rapidly evolving evidence on inclusive pedagogical practices in nursing and midwifery education. This time frame reflects increasing global attention to equity, diversity, and inclusion within health professions education, as well as the emergence of more contemporary approaches to teaching sexual and gender diversity.

#### Exclusion Criteria

Studies will be excluded if they focus exclusively on postregistration education, graduate degree programs, or continuing professional development or if they are opinion pieces or editorials that lack actionable descriptions of pedagogical practices.

### Information Sources

We will search the following databases: CINAHL (EBSCOhost), MEDLINE (via Ovid or PubMed), PubMed (for in‑process and publisher‑supplied records in addition to MEDLINE indexing), Scopus, and Web of Science. Searches will be supplemented by reference list screening of included sources. Preliminary searches (Textbox 1) were conducted in November 2025 to inform strategy development; the final search will be conducted in May 2026. Boolean operators (AND and OR) were used to combine search terms, with truncation (*) applied to capture variations. Quotation marks were used to identify exact phrases (eg, “gender diversity”).

**Literature search strategy and number of records retrieved from each database.**

**Search strategy**

- (Lesbian* OR Gay* OR Bisexual* OR Trans* OR Queer* OR Intersex* OR Asexual* OR LGBT* OR LGBTQIA* OR Two-Spirit) AND (Nurs* OR “Student Nurs*” OR “nurs* student” OR Nurs* and Midwive* OR Midwife*) AND (Education OR Pedagog* OR Teaching OR Learning OR Training)
- AND (Equity OR “Gender Equity” OR “Gender diversity” OR “Sexual Minority” OR “Gender Minority” OR Minority)

**Database and number of results**

- CINAHL: 438
- MEDLINE: 453
- PubMed (includes MEDLINE): 959
- Scopus: 1174
- Web of Science: 776

### Search Strategy

The search strategy was developed iteratively around three concept blocks: (1) sexual and gender diversity, (2) nursing or midwifery students, and (3) equitable education or pedagogy.

### Selection of Sources of Evidence

All search results will be exported to Covidence (Veritas Health Innovation) systematic review software and deduplicated. Two reviewers will independently screen titles and abstracts against the inclusion criteria, followed by full-text screening. During independent screening, disagreements between the two reviewers will be resolved through discussion and consensus. If consensus cannot be reached, a third reviewer (senior researcher) will make the final decision. The number of conflicts and the methods used to resolve them will be recorded for reporting. Reasons for exclusion at the full-text stage will be recorded. The study selection process will be reported using a PRISMA (Preferred Reporting Items for Systematic Reviews and Meta-Analyses) flow diagram [22], detailing the number of records identified, duplicates removed, records screened, full-text articles assessed, and studies excluded to enhance transparency and reproducibility.

The search strategy was developed in accordance with JBI guidance for scoping reviews. Reporting of the completed review will follow the PRISMA-ScR checklist to enhance transparency and completeness of reporting.

### Data Charting and Extraction

#### Overview

The data charting form (Multimedia Appendix 1) was designed to align directly with the review questions. Information relating to pedagogical approaches, intervention characteristics, and implementation strategies will address the primary review question and the first secondary review question. Data concerning theoretical or conceptual frameworks will address the second secondary review question. Information regarding study limitations, recommendations, underrepresented populations, and gaps identified by authors or reviewers will contribute to the third secondary review question and the Patterns, Advances, Gaps, Evidence for Practice, and Research Recommendations (PAGER) synthesis. The data charting form will be pilot-tested on a sample of included studies to ensure consistency and comprehensiveness. The form will be refined iteratively as required. Two reviewers will independently extract data from a subset of included studies to enhance consistency and reliability. Any discrepancies will be resolved through discussion or consultation with a third reviewer.

The data extraction template has been designed to capture detailed information across the domains described in the following sections.

#### Article Characteristics

For each included source, we will extract bibliographic and descriptive information, including the type of article (eg, primary study and description of intervention) and overall study purpose. This will enable classification of the evidence base and differentiation between empirical and descriptive literature.

#### Intervention Design and Pedagogical Approach

We will extract detailed information on the pedagogical approach described, including the type of intervention (eg, simulation, curriculum-based intervention, short-form teaching such as lectures or webinars, educational modules, arts-based approaches, theoretical or pedagogical frameworks, or other formats). Delivery mode (eg, online or virtual, in person, or hybrid) and the presence of theoretical or conceptual underpinnings will also be recorded.

#### Population and Target Audience

Data will be extracted regarding the intended audience of the intervention (eg, preregistration nursing students and preregistration midwifery students). We will also document who delivered the intervention (eg, nursing faculty, midwifery faculty, other educators, or unspecified) to better understand educational roles and responsibilities.

#### Content Focus

The specific sexual and gender diversity content addressed within the intervention will be categorized using predefined labels (eg, transgender, lesbian, gay, bisexual, Two-Spirit, and other identities where specified). This will enable mapping of which populations are included or underrepresented in educational approaches.

#### Study Design and Characteristics

For empirical studies, we will extract methodological details, including study design (eg, qualitative, mixed methods, survey, cohort, cross-sectional, case study, content analysis, or other), study aims, and participant characteristics. Information on the study population (eg, students, educators, clinicians, administrators, or service users), recruitment methods, and sample size will also be recorded where reported.

#### Intervention Description and Evaluation

Where applicable, we will assess whether the intervention is clearly described (eg, fully described, partially described, or not described). A narrative summary of the intervention will be recorded to capture key components, structure, and delivery.

#### Outcomes and Findings

We will extract information on whether outcomes were reported (eg, no outcomes measured, or outcomes assessed using before and after or other evaluation methods). Outcomes will be recorded descriptively and may include knowledge, attitudes, skills, confidence, or changes to the learning environment. General study findings and key results will also be captured.

Educational outcomes will be extracted to provide contextual understanding of how inclusive pedagogical practices have been evaluated within the literature. The review does not seek to determine intervention effectiveness or compare outcomes across studies. Rather, outcome data will be used descriptively to characterize evaluation approaches and identify areas where evidence regarding educational impact is emerging or absent.

#### Contextual Information

Contextual variables will include country of study or intervention, educational setting (eg, university, clinical skills laboratory, and practice environment), and time frame of the study or intervention, where available. This will support analysis of geographical and contextual variation.

#### Data Collection and Analysis

We will extract information regarding data collection methods (eg, surveys, interviews, focus groups, and document analysis) and analytical approaches used. This will support the understanding of how evidence in this field is generated.

#### Synthesis-Relevant Data

To support synthesis using the PAGER framework, we will extract key findings, implications for future research, reported study limitations, and the review team’s analytic notes documenting preliminary interpretations regarding each study’s contribution to the review objectives, including notable strengths, unique contributions, limitations, or emerging evidence gaps. These notes will not be treated as extracted study data but will support reflexive interpretation during synthesis.

#### Additional Notes

Free-text fields will be used to capture additional observations, including any unique features of the study, methodological concerns, or notable contributions not otherwise captured in structured fields.

### Synthesis of Results

Data synthesis will follow the PAGER framework, which was developed specifically to strengthen transparency and methodological rigor in scoping review reporting [23]. The framework provides a structured approach for organizing and interpreting evidence beyond descriptive mapping.

Following data extraction, findings will undergo analysis. Extracted data relating to pedagogical approaches, implementation characteristics, theoretical frameworks, outcomes, contextual factors, and equity considerations will be coded and grouped into themes. Themes will subsequently be organized according to the five PAGER domains: (1) patterns: recurring pedagogical approaches and implementation strategies; (2) advances: innovative or emerging educational practices; (3) gaps: areas where evidence remains limited or absent; (4) evidence for practice: implications for curriculum development, educator preparation, and educational policy; and (5) research recommendations: priorities for future research and evaluation.

Themes will be examined across nursing and midwifery contexts, geographic regions, educational settings, and SGD populations where sufficient evidence exists.

### Ethical Considerations

No ethics approval was required because the review used data from published studies and their findings. No primary research involving individual-level data from human participants was conducted. Dissemination will include publication in a peer-reviewed journal, presentations at conferences, and sharing findings with stakeholders with an interest in this area of research.

## Results

The scoping review was initiated in May 2026 following protocol registration with the OSF. Preliminary searches conducted in November 2025 informed the final search strategy. The final searches were undertaken in May 2026 across 5 databases. At the time of manuscript submission, screening and data extraction were in progress. Data charting and synthesis are expected to be completed by October 2026, with findings anticipated for dissemination in December 2026.

## Discussion

### Expected Findings

This scoping review is expected to identify a diverse range of inclusive pedagogical practices used to address sexual and gender diversity in preregistration nursing and midwifery education. These are likely to include curriculum-based approaches, simulation-based learning, case-based teaching, reflective exercises, and short-form educational interventions (eg, lectures or workshops), delivered across both in-person and online environments.

The review is anticipated to reveal substantial variation in how sexual and gender diversity content is conceptualized and implemented, including differences in the populations represented (eg, emphasis on sexual minority groups compared to gender diverse populations) and the extent to which intersectionality is addressed.

It is expected that many interventions will be underpinned by broad concepts such as cultural competence or cultural humility [16], while fewer studies may explicitly report the use of established pedagogical or theoretical frameworks specific to inclusive or equity-oriented education [24].

In terms of evaluation, the review is likely to identify that outcomes are most commonly focused on short-term measures such as knowledge, attitudes, confidence, and self-reported competence, with limited evidence relating to longer-term impacts on clinical practice or patient outcomes.

The findings are also expected to highlight methodological variability across studies, including differences in study design, sample size, and reporting quality, which may limit comparability. Consistent with the nature of scoping reviews, gaps in evidence are anticipated, particularly in relation to (1) rigorously evaluated educational interventions, (2) inclusion of diverse gender identities (eg, nonbinary and gender diverse populations), (3) representation of midwifery-specific educational contexts, and (4) research conducted outside high-income country settings.

Using the PAGER framework, the review is expected to identify clear patterns in commonly used teaching approaches, advances in innovative or emerging pedagogical practices, gaps in the existing evidence base, implications for educational practice, and priorities for future research.

### Strengths and Limitations

This scoping review will use established methodological guidance from the *JBI Manual for Evidence Synthesis* and will be reported in accordance with the PRISMA-ScR checklist, ensuring transparency and reproducibility. A comprehensive search strategy across multiple bibliographic databases will support the identification of a broad range of evidence relating to inclusive pedagogical practices in preregistration nursing and midwifery education. The use of duplicate screening and data charting will enhance methodological rigor and reduce selection bias. In addition, the application of the PAGER framework will enable structured synthesis of patterns, advances, gaps, and implications for practice and future research. The findings of this review will be relevant to nursing and midwifery educators, curriculum developers, academic leaders, regulators, and policymakers involved in preregistration education. By mapping current pedagogical approaches and identifying gaps, the review will support evidence-informed curriculum design and educator development aimed at improving care for SGD populations.

However, several limitations should be acknowledged. The review team has expertise in nursing and midwifery education and equity-oriented research. Reflexive discussions will be undertaken throughout the review process to minimize bias and to ensure that interpretations remain grounded in evidence. Restricting inclusion to English-language publications may result in the exclusion of relevant international evidence and introduce language bias. As a scoping review, this study will not undertake formal critical appraisal of included sources, and therefore the quality of evidence will not be assessed. Heterogeneity in educational contexts, pedagogical approaches, and outcome measures may limit the comparability of findings and preclude conclusions regarding effectiveness. Finally, the focus on published literature may overlook informal or locally implemented educational practices that are not formally documented.

## Appendix

Multimedia Appendix 1 Data charting template.

Multimedia Appendix 2 PRISMA-ScR checklist.

## Abbreviations

2SLGBTQIA+: Two-Spirit, lesbian, gay, bisexual, transgender, queer or questioning, intersex, asexual, and other sexual and gender identities
JBI: Joanna Briggs Institute
OSF: Open Science Framework
PAGER: Patterns, Advances, Gaps, Evidence for Practice, and Research Recommendations
PRISMA: Preferred Reporting Items for Systematic Reviews and Meta-Analyses
PRISMA-ScR: Preferred Reporting Items for Systematic Reviews and Meta-Analyses Extension for Scoping Reviews
SGD: sexual and gender diverse

## Data Availability

No datasets have been generated or analyzed at the protocol stage. Following completion of the review, extracted data charting tables, search strategies, and supplementary review materials will be made publicly available through the Open Science Framework project associated with this review, subject to journal requirements.
