# Supplementary material for: Addressing Sexual and Gender Diversity Through Inclusive Pedagogical Practices in Global Preregistration Nursing and Midwifery Education: Protocol for a Scoping Review
Source: JMIR Res Protoc. 2026 Sep 14;15:e93408. doi: 10.2196/93408 (PMC13620429; doi:10.2196/93408)
Supplement: Supplementary file 1 [file resprot_v15i1e93408_app1.docx]

| Domain | Data Items Extracted |
| --- | --- |
| Article Characteristics | Author(s); year; title; country; article type (primary study, intervention description, evidence synthesis, grey literature, other); overall study purpose/aim |
| Intervention Design and Pedagogical Approach | Type of pedagogical approach (simulation, curriculum intervention, lecture/workshop, educational module, case-based learning, reflective learning activity, arts-based approach, assessment strategy, faculty development, other); delivery mode (online, in-person, hybrid); intervention duration; mandatory or optional participation; stand-alone or embedded curriculum component; theoretical or pedagogical framework; description of framework application |
| Population and Target Audience | Intended audience; intervention facilitator(s); participant characteristics; sample size |
| Content Focus | Sexual and gender diversity populations addressed; description of content focus; equity and intersectionality considerations |
| Study Design and Characteristics | Study design; study aims; participant characteristics; recruitment methods; sample size |
| Intervention Description and Evaluation | Description of intervention; intervention components; intervention structure; clarity of intervention reporting; narrative summary of implementation and evaluation approach |
| Outcomes and Findings | Whether outcomes were reported; outcome measures; knowledge, attitudes, skills/competence, confidence, behaviour change, learning environment, other outcomes; key findings and results |
| Contextual Information | Country; educational setting; programme type; timeframe/duration of intervention |
| Data Collection and Analysis | Data collection methods; data analysis methods; methodological notes |
| Synthesis-Relevant Data | Key findings relevant to review questions; implications for educational practice; implications for future research; reported study limitations; reviewer analytic notes; PAGER coding (Patterns, Advances, Gaps, Evidence for Practice, Research Recommendations) |

Note: The data charting form was developed iteratively and will be pilot-tested on a sample of included studies. Modifications may be made during the review process to ensure comprehensive capture of data relevant to the review objectives and planned synthesis using the PAGER framework. The sections related to PAGER will be completed once the full texts have been extracted and consensus agreed.
